# Delayed diagnosis of cluster headache in Portugal

**DOI:** 10.1055/s-0046-1827040

**Published:** 2026-08-31

**Authors:** Lénia Silva, Tiago Millner, Mariana Cabral, Aurora Costa, Pedro Almeida, Beatriz Barreto, Madalena Pinto, Rafael Dias, Stefanie Moreira, Gonçalo V. Bonifácio, Sofia Marinho Pinto, Rita Castro, Ivânia Alves, Ana Luísa Rocha, Sara Varanda, Andreia Costa, Carlos Andrade

**Affiliations:** 1 Unidade Local de Saúde de Santo António, Departamento de Neurologia, Porto, Portugal.; 2 Unidade Local de Saúde de São João, Departamento de Neurologia, Porto, Portugal.; 3 Hospital Central do Funchal, Departamento de Neurologia, Funchal, Madeira, Portugal.; 4 Hospital Divino Espírito Santo, Departamento de Neurologia, Ponta Delgada, Açores, Portugal.; 5 Unidade Local de Saúde de Braga, Departamento de Neurologia, Braga, Portugal.; 6 Unidade Local de Saúde de Vila Nova de Gaia/Espinho, Departamento de Neurologia, Vila Nova de Gaia, Portugal.; 7 Universidade do Porto, Faculdade de Medicina, Departamento de Neurociências Clínicas e Saúde Mental, Porto, Portugal.; 8 Unidade Local de Saúde de Arrábida, Departamento de Neurologia, Setúbal, Portugal.; 9 Unidade Local de Saúde de Entre Douro e Vouga, Departamento de Neurologia, Santa Maria da Feira, Portugal.

**Keywords:** Cluster Headache, Delayed Diagnosis, Age at Onset

## Abstract

**Background:**

Cluster headache (CH) has well-defined diagnostic criteria, but its diagnostic delay (DD) remains substantial, averaging 3.6 to 9 years, leading to inappropriate treatments and significant psychosocial and socioeconomic burdens.

**Objective:**

To identify demographic and clinical factors associated with DD in CH to promote earlier diagnosis.

**Methods:**

A multicenter cross-sectional study recruited 64 patients with episodic or chronic CH from 6 Portuguese neurology centers. Participants completed an online questionnaire covering demographics, clinical features, prior consultations, diagnoses, and treatments. Diagnostic delay was defined as the time from symptom onset to formal diagnosis. Correlation tests and multiple linear regression were performed to identify predictors of DD.

**Results:**

The mean DD was of 6.4 ± 8.3 (range: 0–39) years, with 46.9% experiencing delays > 4 years. No sex differences were observed. Younger age at onset (ρ = –0.45; *p* < 0.001); attack onset between midnight and 3 am ( *p* = 0.029), and eyelid edema ( *p* = 0.001) were associated with shorter DD. The number of pain episodes before neurology consultation strongly correlated with DD (ρ = 0.92; *p* < 0.001), underscoring the impact of referral delays. Most patients (59.4%) received alternative diagnoses, mainly migraine and sinusitis. Prior triptan ( *p* = 0.037) and topiramate use ( *p* = 0.012) were linked to longer DD. Multivariate models highlighted age at onset and clinical features as primary predictors, explaining nearly 60% of variance.

**Conclusion:**

Diagnostic delay in CH remains considerable in Portugal. Increasing awareness among primary care physicians and non-neurologist specialists is crucial to reduce DD and inappropriate treatments. Further research should clarify the role of educational level and support targeted interventions for timely recognition.

## INTRODUCTION

Cluster headache (CH) is a rare primary headache disorder classified as a trigeminal autonomic cephalalgia. It is characterized by recurrent attacks of severe unilateral headache, typically accompanied by ipsilateral cranial autonomic symptoms and/or restlessness. 1 Attacks last between 15 and 180 minutes and may occur from once every other day up to 8 times per day during active periods, which usually span several weeks. 2 Cluster headache affects approximately 0.1% of the general population and is more prevalent in men, with peak onset between the ages of 20 and 30. 2 3 4 A circadian pattern, predominantly nocturnal, and a circannual rhythm are typical features of the disorder. In the absence of specific biomarkers, CH is diagnosed clinically, based on a detailed medical history. Despite the existence of well-defined diagnostic criteria, diagnostic delay (DD) remains significant, with the time from symptom onset to diagnosis ranging from 3.6 to 9.0 years on average. 5 This delay contributes to misinformation and the prescription of inappropriate, sometimes invasive, treatments. The psychosocial burden of CH is considerable: an American study reported that during attacks, 55% of participants experienced suicidal ideation and 50% engaged in self-harming behavior. 2 The socioeconomic impact is also relevant due to frequent medical leave, work absenteeism, and increased use of healthcare resources. Given the high level of disability associated with CH, early diagnosis and effective treatment are crucial to reducing the disease's impact and preserving quality of life. Identifying predictors of DD and understanding their clinical consequences are essential steps toward minimizing this delay. There are no recent epidemiological studies on CH in Portugal, making it crucial to characterize this population. The present study aims to evaluate the DD in CH, identify associated demographic and clinical factors, enable comparisons with other countries, and provide a deeper understanding of the national context, thereby informing future health and policy measures.

## METHODS

The current study included patients diagnosed with episodic or chronic CH according to the criteria established by the International Classification of Headache Disorders, 3rd edition (ICHD-3). 1 Participants were recruited from the outpatient Neurology centers of six public hospitals in Portugal. Between September 2024 and March 2025, patients were consecutively invited to complete an online questionnaire. Access to the questionnaire was granted only after reading and accepting the informed consent presented on the form's introductory page. The questionnaire ( **Supplementary Material** – available at https://www.arquivosdeneuropsiquiatria.org/wp-content/uploads/2026/04/ANP-2025.0405-Supplementary-Material.pdf ), developed by the authors, included demographic data, information on the medical specialties consulted before the diagnosis of CH, dates of the first headache attack and formal diagnosis, diagnoses received, and treatments prescribed before the correct diagnosis. Triptan use was recorded as a general category, without distinction between routes of administration (e.g., oral, subcutaneous, or intranasal). The data were compiled into a database and analyzed using IBM SPSS Statistics for Windows (IBM Corp.), version 29.0. The study protocol was approved by the Ethics Committees of all participating hospitals.

### Statistical analysis

Data are presented as numbers and percentages, means with standard deviation (SD), or medians with interquartile range (IQR), as appropriate. The normality of each variable was assessed using the Shapiro-Wilk test due to the small sample size. Diagnostic delay (months between symptom onset and diagnosis) was defined as the main outcome variable and was assessed using retrospectively self-reported data at a single time point. Its initial distribution was highly skewed (mean 84.55 ± 103.69; median 44.00), with elevated skewness (1.90 ± 0.30) and kurtosis (4.04 ± 0.59) indicating non-normality. Given the lack of prospective follow-up or censoring, formal survival analysis was not applied. To fulfill assumptions for parametric testing, a logarithmic transformation was applied, allowing a more symmetric distribution (mean 1.50 ± 0.75; median 1.65) with reduced skew (–0.61 ± 0.30). Consequently, regression coefficients in linear models represent multiplicative associations with DD (i.e., percent change per unit increase in the predictor) rather than absolute differences in months. Exponentiated coefficients (exp[β]) are reported for interpretability.

An independent samples t-test or the Mann-Whitney U test was used to compare continuous variables between categorical groups, depending on data normality. For comparisons between categorical variables, Pearson's Chi-squared test was applied. The association between two quantitative variables was assessed using Pearson or Spearman correlation coefficients, depending on data distribution.

To study the factors that independently contribute to DD in CH, several multiple linear regression models were constructed. Each model grouped variables with similar characteristics (sociodemographic, clinical, symptomatic, and therapeutic) to reduce multicollinearity and identify the most statistically robust predictors. Age at diagnosis was excluded from all models to avoid structural collinearity with DD, and age at symptom onset was retained as a clinically meaningful predictor. A *p* -value < 0.05 was considered statistically significant.

## RESULTS

Of the 100 patients contacted, 64 completed the questionnaire, yielding a response rate of 64%. Among respondents, 53 (82.8%) were male, resulting in a male-to-female ratio of 4.8. The mean age at the time of the study was 49 ± 12.5 years. The mean age at the first headache episode was 34.2 ± 12.2 years, and the mean age at diagnosis was 40.6 ± 11.4 years. The DD was 6.4 ± 8.3 (range: 0–39) years. A total of 30 patients (46.9%) experienced a DD exceeding 4 years.

Episodic CH was identified in 44 participants (68.8%), and only 2 participants had a family history of CH. Twenty-four (37.5%) participants had a university-level education. Regarding lifestyle habits, 36 (56.3%) were smokers, 17 (26.6%) consumed alcoholic beverages, and 28 (43.8%) engaged in physical exercise. Most patients reported photophobia (64.1%) and phonophobia (53.1%) during headache attacks. Only 4 patients (6.3%) denied experiencing cranial autonomic symptoms during episodes. Restlessness and anxiety were frequently reported, affecting 78.1% and 70.3% of respondents, respectively. The most frequent time of attacks was during the early nocturnal hours (0–3 am) in 20 patients (31.3%), followed by the late-night hours (3–7 am) in 19 (29.7%), in the morning (7 am–2 pm) in 6 (9.4%), in the afternoon (2–7 pm) in 9 (14.1%), and in the evening (7 pm–12 am) in 10 patients (15.6%).

Sex had no significant effect on DD ( *p* = 0.887). Patients with higher schooling levels (bachelor's or master's/doctoral degrees) tended to have longer DD compared to those with lower education (although this difference did not reach statistical significance, 8.4 vs. 2.3 years; *p* = 0.065). Similarly, a non-significant but potentially clinically relevant trend toward shorter DD was observed in patients who engaged in regular physical exercise ( *p* = 0.175; Cohen's d > 0.7). Younger age at symptom onset was significantly associated with a shorter DD (ρ = -0.45; *p* < 0.001). Eyelid edema (exp[β] = 0.51; *p* < 0.001) and early nocturnal attack timing (0–3 am; exp[β] = 0.49; *p* = 0.011) were associated with shorter DD, while afternoon attacks predicted longer DD (exp[β] = 1.92; *p* = 0.006). Cluster headache type, pain duration and intensity, the presence of autonomic symptoms, restlessness or anxiety, and the number of specialists consulted before diagnosis were not individually associated with DD.

The first medical specialty consulted following disease onset was General Practitioner (34%), followed by Neurology (25%), Emergency Services (22%), Dentistry (11%), Ophthalmology (5%), Neurosurgery, and Otorhinolaryngology (both 1.6%). Before receiving a definitive diagnosis, 57.8% of patients had consulted two or more different specialties, with a median of 2 specialties (range: 1–5). No association was found between patient age and the number of medical specialties consulted before the diagnosis of CH (ρ = 0.083; *p* = 0.517). Consulting specialties other than Neurology was not, in itself, a significant predictor of DD ( *p* = 0.100). However, the strong correlation between the number of pain episodes before Neurology consultation and DD (ρ = 0.92; *p* < 0.001) likely reflects time accumulation, as a longer DD inherently allows for a greater number of episodes, rather than indicating an independent predictive relationship.

Before receiving the final diagnosis, 38 participants (59.4%) had been given alternative diagnoses, with 17 (26.6%) receiving more than 1. The most common initial misdiagnosis was migraine (15 participants, 23.4%), followed by sinusitis (n = 8; 12.5%), trigeminal neuralgia (n = 5; 7.8%), dental problems (n = 4; 6.3%), intracranial hypertension (n = 2; 3.1%), ophthalmologic conditions (n = 2; 3.1%), tension-type headache (n = 1; 1.6%), and psychological causes (n = 1; 1.6%). Patients who received alternative diagnoses demonstrated a non-significant trend toward longer DD ( *p* = 0.103), although the effect size was large (Cohen's d > 0.7).

Before the diagnosis of CH, multiple acute treatments were prescribed: paracetamol (56.3%), non-steroidal antiinflammatory drugs (54.7%), triptans (28.1%), treatments for sinusitis (25.0%), opioids (17.2%), dental procedures (14.1%), and alternative therapies such as acupuncture (9.4%). Preventive treatments were initiated in 43.8% of patients, most commonly antidepressants (20.0%) and antiepileptic drugs (19.0%). A later CH diagnosis was significantly associated with prior treatment with triptans ( *p* = 0.037) and topiramate ( *p* = 0.012). Use of any preventive medication showed a trend towards longer DD ( *p* = 0.074), particularly amitriptyline ( *p* = 0.069).

Multiple linear regression models were constructed to identify factors independently associated with DD. Variables were grouped conceptually (sociodemographic, clinical, symptomatic, and therapeutic) to reduce multicollinearity ( Table 1 ).

**Table 1:** Multiple linear regression models

Model	Included variables	Main results	Exp(β)	*p* -value	Adjusted R ^2^	Overall significance ( *p* -value)
**Sociodemographic**	Sex; age; university attendance; physical exercise	No significant predictors	–	–	0.032	0.208
**Autonomic and neurovegetative**	Lacrimation; ptosis; eyelid edema; photophobia; phonophobia; restlessness; anxiety	Eyelid edema: shorter DD	0.51	< 0.001	0.145	0.025
**Timing (time of day)**	Afternoon attacks (02:00–7:00 pm); early nocturnal attacks (12 am–03:00 am)	Afternoon attacks: longer DD; early nocturnal attacks: shorter DD	Afternoon attacks: 1.92; early nocturnal attacks: 0.49	0.006 0.011	0.145	0.006
**Pain and misdiagnosis**	Pain intensity; attack duration; previous misdiagnoses	No significant predictors	–	–	0.028	0.238
**Therapeutic**	Prior triptan use	Prior triptan use: longer DD	1.70	0.019	0.105	0.052
**Strong clinical model**	Age at symptom onset; number of episodes before consultation; prior incorrect diagnoses	Age at onset: Shorter DD; other predictors not significant	Onset: 0.94	< 0.001	0.597	< 0.001

Abbreviation: DD, diagnostic delay.

Notes: Coefficients are exponentiated (exp[β]) to indicate the multiplicative change in DD per unit increase in the predictor. The *p* -values indicate statistical significance of the individual predictors; overall model significance reflects the F-test for the model.

The sociodemographic model revealed no statistically significant predictors of DD (adjusted R ^2^ = 0.032; p = 0.208). In the autonomic and neurovegetative symptoms model, the presence of eyelid edema was associated with shorter DD (exp[β] = 0.51; *p* < 0.001). Regarding the timing model, afternoon attacks were associated with longer DD (exp[β] = 1.92; *p* = 0.006), whereas early nocturnal attacks were linked to shorter DD (exp[β] = 0.49; *p* = 0.011). The overall model was statistically significant ( *p* = 0.006). A separate model examining pain intensity, attack duration, and prior misdiagnoses did not identify any significant predictors (adjusted R ^2^ = 0.028; *p* = 0.238). Regarding treatment-related factors, only prior triptan use was significantly associated with longer DD (exp[β] = 1.70; *p* = 0.019), with the model approaching overall significance (adjusted R ^2^ = 0.105; *p* = 0.052). Finally, in the strong clinical model, younger age at symptom onset was significantly associated with shorter DD (exp[β] = 0.94; *p* < 0.001), while other variables did not reach statistical significance. This model showed the highest explanatory power among the conceptual models (adjusted R ^2^ = 0.597; *p* < 0.001).

## DISCUSSION

Our study demonstrates a striking DD in CH, with a mean of 6.4 years and extremes of up to 39 years, highlighting that almost half of patients waited more than 4 years for diagnosis. These findings underscore the persistent and clinically significant challenge of timely CH recognition. Comparable international studies have reported similar delays. Klapper et al. found an average DD of 6.6 years in a U.S. cohort, a German study reported a delay of approximately 3 years, a Spanish investigation found an average delay of 4.9 years. 6 7 8 Additionally, a median DD of 3 years (range 1–48) in the Netherlands and an average DD of 3.7 years (range 0–40) in Belgium have been documented, highlighting the widespread nature of this diagnostic challenge. 7 9

The overall demographic profile (high male-to-female ratio and mean age at the first headache episode around thirties) is consistent with previous epidemiological data. 2 3 Our data confirm the presence of classical cranial autonomic symptoms in nearly all patients, with eyelid edema emerging as a particularly strong clinical marker linked to shorter DD. This finding supports previous observations emphasizing the importance of autonomic signs in raising clinical suspicion for CH. 10 11 Notably, about one fifth of the cohort did not report restlessness. However, this variable was not associated with either prolonged or shorter DD in the current study.

Interestingly, sociodemographic factors alone did not significantly explain DD, corroborating Bahra et al. and emphasizing the multifactorial and complex nature of diagnostic barriers beyond patient characteristics. 12 Paradoxically, higher education was associated with longer DD. This finding should be interpreted with caution, as it may reflect multiple factors, including differences in healthcare access, health-seeking behavior, or the use of private versus public healthcare pathways, rather than a direct causal relationship. Further studies are needed to better understand this association. 13

Nonetheless, younger age at symptom onset correlated with shorter DD, suggesting that CH in younger individuals might be more readily recognized. This finding is consistent with the fact that CH typically begins at a younger age. In contrast, several previous studies have reported the opposite trend, with faster diagnosis in older patients. 5 This has often been attributed to the concern that older individuals presenting with headaches may be more promptly referred to specialized care due to the suspicion of secondary causes. However, these findings may suggest greater awareness of the typical age of onset, which could contribute to earlier recognition and diagnosis of CH in younger patients. These results underscore the clinical challenge of recognizing CH across age groups and the need for increased awareness, especially among older patients. The final multivariate model accounted for nearly 60% of the variance in DD, highlighting age at symptom onset and diagnosis as primary predictors. Age at diagnosis was excluded from the model to avoid structural collinearity, ensuring more interpretable and reliable estimates. These findings emphasize that younger age at symptom onset is associated with earlier recognition of CH, independent of age at diagnosis.

The timing of headache attacks was another pivotal factor influencing diagnostic speed. Attacks occurring at early nocturnal hours, a classic cluster period, were associated with earlier diagnosis, whereas afternoon-predominant attacks correlated with longer delays. 2 This may reflect greater familiarity with the nocturnal phenotype of CH and a tendency to misclassify afternoon headaches as tension-type or sinus-related pain, consistent with prior reports highlighting phenotypic variability as a contributor to misdiagnosis. 5 7

The heterogeneity in the first specialties consulted reinforces the lack of recognition of CH outside the context of Neurology, with one third of patients initially seeking General Practice, where early diagnosis may be more difficult due to less familiarity with rare headache disorders. On the other hand, it is mainly from General Practice that patients are referred to hospital Neurology consultations in Portugal. Although no statistically significant differences were found between the non-Neurology specialties consulted before final diagnosis and DD, the number of previous pain episodes was a predictor of longer time to the final diagnosis, emphasizing the importance of early specialist referral to reduce diagnostic latency. This strong association should be interpreted with caution, as it likely reflects the cumulative nature of time rather than a causal or independent predictive effect. The high rate of alternative diagnoses received before confirmation of CH highlights the initial nonspecificity of symptoms and overlap with other primary headaches, particularly migraine. This may lead to the use of inappropriate and ineffective treatments, as well as expose patients to unnecessary risks. Despite some patients receiving multiple alternative diagnoses, this factor did not independently predict delay in our regression models, though a clinical trend was observed. Prior triptan and topiramate use was linked to increased DD, likely reflecting initial misdiagnosis as migraine. As the route of administration of triptans was not specified, some formulations may reflect partial recognition of CH, which should be considered when interpreting these findings. This echoes findings from Klapper et al., who reported frequent misdiagnoses leading to inappropriate therapies and diagnostic postponement. 6

The present study has limitations inherent to its observational design, the use of questionnaires as a data collection method, and potential recall bias. The relatively small sample size and a response rate of 64% may introduce selection and response biases, as patients who respond are often more informed and have greater access to digital resources. In addition, there is a risk of overfitting in the regression models, and the structural relationship between age-related variables required careful consideration, which has now been addressed in the revised models. Nevertheless, the decision to apply the questionnaire was driven by the feasibility of including a larger sample from multiple hospitals at no cost and with greater ease. The multicenter design and use of standardized ICHD-3 criteria strengthen the validity of our findings. Future studies with larger sample sizes and longitudinal designs may further advance the understanding of determinants of DD in CH.

Our findings have practical implications. Earlier recognition and referral of CH may be achieved through improved education of primary care providers and non-neurologist specialists on its clinical hallmarks, particularly restlessness, attack duration and intensity. Additionally, cautious use of empirical therapies before a confirmed diagnosis may prevent masking clinical patterns and reduce delay. The comorbidity burden in patients with CH is well documented, and addressing these comorbidities may help reduce overall disease burden. 14 Moreover, the negative impact of CH on quality of life and response to preventive treatment has been reported in multiple studies, highlighting the importance of timely and targeted interventions. 2 Further research is warranted to clarify sociodemographic influences on DD and to develop strategies to minimize this persistent challenge.

In conclusion, the current study offers valuable epidemiological and clinical insights into the factors that influence CH diagnosis in Portugal. A substantial DD in CH was confirmed within a Portuguese cohort, averaging 6.4 years. Younger age at symptom onset, nocturnal attack timing, and the presence of eyelid edema were associated with earlier diagnosis, while higher education level showed a paradoxical trend toward longer delay. Enhanced awareness of characteristic clinical features and optimized referral pathways are critical to reducing the substantial diagnostic latency that continues to burden patients with CH.
